# Supplementary material for: A cloud-based toolbox for the versatile environmental annotation of biodiversity data
Source: PLoS Biol. 2021 Nov 15;19(11):e3001460. doi: 10.1371/journal.pbio.3001460 (PMC8629388; doi:10.1371/journal.pbio.3001460)
Supplement: Attachment — Submitted filename: Annotator_paper_revisions_responses2 - WJ.docx [file pbio.3001460.s002.docx]

Response to Reviewers

Reviewer #1:
[identifies himself as Christopher Anderson]

In this work, Li et al. describe a new cloud-based system for annotating spatially-explicit species occurrence records and range map data with multi-scale, remotely-sensed environmental data. STOAT provides a browser-based UI and an open source R package, making this work accessible to a range of user expertise, and supports the analysis of custom user datasets. This system was designed to overcome several of the issues introduced by spatial and temporal scaling dynamics that affect nearly all analyses of ecological data by helping users explore a range of spatiotemporal buffering options. It is likely to become an important tool for the ecological research and biodiversity monitoring communities, and I commend the authors and developers. At this point I believe there are a few key edits to the manuscript that the authors should make in order to clarify some important issues for readers. These relate to the terminology used throughout, the potential uses of this system, and a reconsideration of the spatial scaling analysis.

***We thank Dr. Anderson for the very thorough and helpful review. We hope our revisions successfully address they key questions and suggestions***

First, I think it will be critical for the authors to clearly define what "annotation" refers to. I read the manuscript all the way through the first time without every discovering what it meant. It only became clear to me after I visited the R package README and read through the Github repository. It is a clear and appropriate term once the reader understands what it means, but it is not in common usage (at least within the literature I'm familiar with). I suppose I colloquially refer to it as feature extraction, or simply as zonal statistics, but these don't quite address the more comprehensive process developed for STOAT.

***We agree that a clearer and more explicit definition of “environmental annotation” is important. We have added this definition to the introduction (track-changes manuscript line 52) ***

I believe there is an opportunity for the authors to clearly define biodiversity data annotation, to state why it is important to explore data that has been annotated with multi-scale features, and to discuss the potential insights to be gleaned that will improve our understanding of biodiversity change.

***We agree, and have added a section on the advantages of conducting scale-conscious environmental annotations (line 164). Within this section we highlight spatiotemporal buffers as a tool for conducting multi-scale annotations, and discuss the twofold advantages of conducting multi-scale annotations: 1) to improve the overall spatiotemporal environmental characterizations of species for analyses that may require species-environment matching, and 2) to explore and quantify scaling relationships themselves. We refer readers to our case studies, which represent use cases for STOAT’s scale-adjustable environmental annotations. ***

The manuscript includes a strong introduction to the challenges posed by common and beguiling problems related to pattern and scale in ecology, and STOAT addresses many of them from an interesting conceptual and technological standpoint. In that sense, what the system does is clearly described. Yet it wasn't clear to me how these annotated data would be used. I had neither a sense for what other analyses these annotations would support (e.g. are these features to be included in species distribution models?) nor how the annotated data could be exported or used (the web tool appears, at the moment, to just show maps and graphs). I can imagine a few potential uses of these data - and I'm sure the authors have imagined more - but my sense was that the paper focuses more on describing the technical challenges it overcomes and less on how overcoming those challenges addresses broader problems for the community. I recommend balancing these topics.

***We agree with this point. We hope that the newly added paragraph mentioned above addresses these concerns to an extent with its explicit discussion of improved/new types of analyses enabled by STOAT. For more specific use cases, we now refer readers to our case studies, which demonstrate how STOAT’s buffers can be used to quantify 1) scaling relationships in biodiversity-environmental characterizations (Figures 6-7), and 2) differences in environmental characterizations, compared to ‘normal’ annotation methods (new Figure 8). These case studies additionally demonstrate example workflows that incorporate STOAT and its annotation capability. We hope that the addition of the third case study brings further detail to STOAT’s potential uses***

Finally, I recommend reconsidering or reanalyzing the section characterizing variation across spatial grains; one of the few quantitative contributions of this manuscript. The proposed analysis (evaluating shifts in vegetation growth patterns (here, EVI) across spatial scales to quantify how species-environment relationships change with buffer size) is interesting and worth performing. However, from what I can tell, the author's analysis introduced a major source of variation: comparing EVI from two separate satellite sources. I suspect this is actually driving the results as presented, and needs to be addressed in a revision. There are a few sources of variation I'd like to point out.

***We thank Dr. Anderson for his concern regarding the combination of data from differing satellite sources. However, we believe that there has been a misunderstanding regarding this EVI case study. The case study did not combine MODIS and Landsat EVI, rather it used Landsat 8 EVI data from a single source throughout. The two scales of data assessed in the original analysis, 30m and 250m, were both using the 30m Landsat 8 EVI layer, and it was STOAT’s spatial buffer that was used to coarsen the 30m grain annotation to 250m. We understand the confusion, as MODIS data were also used in the case studies, but strictly for Land Surface Temperature, not EVI. We realize that a lack of clarity in the manuscript may have contributed to this misunderstanding, and have made some textual revisions (line 382 on) to improve clarity and specifically indicate that the EVI data were all Landsat 8 (line 492) ***

- It is not easy to directly compare Landsat and MODIS EVI calculations, even when using harmonized ARD products. Beyond differences in radiometric calibration, signal-to-noise ratios, wavelength centers, spectral resolution and sensor-specific EVI coefficients, a major difference is in the native spatial resolution of each sensor. Due to complex radiative transfer processes, the at-sensor radiance patterns measured by a satellite changes non-linearly as the grain size of a sensor increases. In other words, resampling a group of 30x30 m measurements to 250x250 m grid cells will not necessarily match a direct 250x250m measurement over the same area. This introduces a systematic bias that is not addressed.

***This point is addressed by the preceding response***

- The spatial uncertainty assumed by the authors (~100 m) is greater than the grain size of the fine scale measurements (30 m), meaning it's likely that many observations annotated with nearest-neighbor data are not precisely labeled. This is probably fine to a degree (first law of geography and such), and would probably average out with enough samples, but is likely to introduce a broad distribution of residuals that make it look like there's more disagreement than there really is. This is what I see when I look at the Delta EVI histogram in Figure 6.

***We fully agree that this was an issue with the previous EVI case study, and have thus rerun the Figure 6 analysis using spatial buffers of 120m vs. 990m to stay at the scale of estimated uncertainty, as opposed to the previous 30m vs. 250m comparison. We found similar results to that of the 30 vs. 250m comparison, and have updated numbers in the case study***

- Oh, and there are (likely small) differences in horizontal positional accuracy (i.e. pixel location uncertainty), as well as differences introduced by the spatial projections of the data processing streams (WRS-2 for Landsat, Sinusoidal for MODIS) that would further balloon residual distributions.

***This point is also addressed by the first response on this topic above, that only one remote sensing data source is used***

I did not feel the temporal grain analysis suffered from the same issue, however, as they analyzed a consistent data source.

I recommend running a similar analysis that isolates differences between grain sizes by analyzing data that's been resampled from fine to coarse scales from a single sensor (e.g. resampling 30 m -> 240 m Landsat EVI (as the closest round multiple to 250 m)). This would obviate the differences introduced by radiometric calibration differences / etc. and create a comparison that isolates scale effects. I would probably be willing to let the spatial uncertainty issue slide if this analysis were done well and if the issue was clearly addressed in the text as a caveat.

***This was indeed the methodology of the original analysis, although through the use of STOAT’s scale-adjustable spatial buffers instead of adjusting the resolution of the environmental layer directly. We note Anderson’s consideration of using round multiples of the native 30-meter data grain for our analysis, and therefore use round multiples of 30 meters in our reanalysis (120 vs 990 meters) ***

The authors have developed a valuable cloud-based tool that I expect will be received well by the community. After revisions, I suspect this  manuscript will be as well.

***Thank you ***

Below are some minor notes that don't need to be specifically addressed, but may be useful feedback for the authors.

- The R package page and the Github repository are not linked in the paper. It seems like they should be.

***We have added the R package CRAN page to the paper on line 237, adjacent to the Github repository link for rstoat***

- Based on exploring the web UI, it looks like STOAT is optimized for working with GBIF data. I think the authors would do well to explicitly call that out to orient the reader to which ecosystem of tools this system fits into (outside of the obvious MoL connection).

***We have added a mention of GBIF to the paragraph: “STOAT functionality and usage,” on line 242***

- GBIF has an API, right? It seems a future version could just hook up to that instead of having users manually filter, download the csv files, then trim the fields to prepare for upload. I found this manual process to be a bit arduous and confusing (e.g., in the field reconciliation process).

***We agree that the data upload process could be streamlined in the future and appreciate this comment. We do have plans to link STOAT more directly with upstream biodiversity data repositories, primarily in the R environment***

- Requiring (instead of making optional) a license for uploads seems like the wrong choice: I suspect the majority of users will come here with GBIF records, which they do not have the right to assign a license to. Seems like there should be more flexibility here.

***We note that the licensing requirements are primarily in place to better work with Map of Life’s data partners, including GBIF. The licensing requirements help to ensure that all datasets published on Map of Life have proper attribution and are owned by their respective uploaders, discouraging arbitrary publication of unowned data. If users upload GBIF data for personal use in STOAT, they are expected to make the dataset private, in which case the license does not actually apply, as the dataset is completely invisible***

- Assuming 100 m spatial accuracy for records seems generous, but of course will vary based on the source (cell phones vs. museum records). The authors discuss this as an issue, but how it effects the interpretation of results could be more clear.

***We appreciate Dr. Anderson’s concern regarding the spatial accuracy of the occurrence records. We agree that spatial accuracy depends on the data source, but note that for the time period (2013-2017) assessed, we do not expect to see any museum records due to the overwhelming abundance of citizen science records in eBird. Rather, the data are dominated by backyard birdfeeder occurrences, which are pinned by users on the map, and which we expect to fall within our 100m estimated spatial uncertainty. We thus don’t believe that the spatial uncertainty issue is affecting our results significantly***

- The discussion of the EVI differences is hard to interpret. The range of values is relatively small, and not tied to any directly interpretable biophysical pattern. I felt I could use more guidance as a reader in how to understand the meaning of a median difference of 0.06 EVI.

***We agree, and have thus added some context, saying that the mean EVI of Northeastern U.S. mixed forests (inhabited by both the ruby-throated hummingbird and the tufted titmouse) ranges annually from approximately 0.2 in the winter to 0.6 in the summer***

- The last paragraph of the introduction to the discussion (l. 436-441) could be fleshed out more - it's a bit vague.

***We agree with this comment, and have added substantially more detail to that paragraph***

Reviewer #2:

I enjoyed the introductory section entitled Matching the respective scales of observations, uncertainty, and process.  It was an excellent review in clear language without jargon, but a bit duplicative.

***We thank the reviewer for their helpful comments. We have trimmed parts of the text in this section to reduce the wordiness***

This section had me expecting that STOAT would allow integration of data with different spatiotemporal attributes with the environmental data layers but this is not the its function. Perhaps that can be modeled into future products to make use of more data for species with less data. STOAT seeks to properly match the spatiotemporal attributes of the biodiversity spatial data and the environmental data layers.  This is important and if not done can lead to misinterpretation as stated by the authors. Any solution that makes data annotation for fitness for use easier is welcomed and STOAT appears to work well. The discussion provides realistic expectation of the data analysis which is appreciated. There is no overselling of the procedure.

***We thank the reviewer. We intend for STOAT to serve both data fusion purposes, i.e., 1) to integrate biodiversity data with differing attributes, and 2) to match biodiversity data scale with that of environmental layers. The newly added case study directly deals with data of heterogeneous spatial grains, and we hope it elucidates some of the other use cases for STOAT that might not have been as immediately apparent previously***

My main question is: What would the data for the two case studies look like if it had not been filtered through STOAT?  There is no comparison on the two case studies to a "dirty" analysis with raw data.  What results would that give?  This could be a strong selling point for the paper.

***We agree that this was a limitation of the previous manuscript, and we have added a new case study and analysis that directly seeks to answer this question. We compare STOAT’s record grain-specific annotation methodology to a simpler and more commonly-used record grain-agnostic 1km annotation, and find significant differences in the resultant EVI characterizations in both the spatial and temporal dimensions. We hope that this addition addresses the reviewer’s question.***
